# Association of microRNA Expression and BRAF^V600E^ Mutation with Recurrence of Thyroid Cancer

**DOI:** 10.3390/biom10040625

**Published:** 2020-04-17

**Authors:** Daina Pamedytyte, Vaida Simanaviciene, Dalia Dauksiene, Enrika Leipute, Aurelija Zvirbliene, Valdas Sarauskas, Albertas Dauksa, Rasa Verkauskiene, Birute Zilaitiene

**Affiliations:** 1 Institute of Biotechnology, Life Sciences Center, Vilnius University, LT- 10257, Sauletekio av. 7, Vilnius, Lithuania; daina.pamedytyte@gmc.vu.lt (D.P.); vaida.simanav@gmail.com (V.S.); lei.enrika@gmail.com (E.L.); azvirb@ibt.lt (A.Z.); 2 Institute of Endocrinology, Medical Academy, Lithuanian University of Health Sciences, LT-50161 Eiveniu str. 2, Kaunas, Lithuania; dalia.dauksiene@gmail.com (D.D.); rasa.verkauskiene@lsmuni.lt (R.V.); 3 Department of Pathology, Lithuanian University of Health Sciences, LT-50161 Eiveniu str. 2, Kaunas, Lithuania; sarauskasvaldas@yahoo.com; 4 Institute for Digestive Research, Medical Academy, Lithuanian University of Health Sciences, LT-50161 Eiveniu str. 2, Kaunas, Lithuania; albertas.dauksa@gmail.com

**Keywords:** papillary thyroid carcinoma (PTC), miRNA, BRAF^V600E^ mutation, recurrence, biomarkers

## Abstract

Many miRNAs and cancer-related mutations have been proposed as promising molecular markers of papillary thyroid carcinoma (PTC). However, there are limited data on the correlation between miRNA expression, BRAF^V600E^ mutation, and PTC recurrence. Therefore, to evaluate the potential of BRAF^V600E^ mutation and five selected miRNAs (-146b, -222, -21, -221, -181b) in predicting PTC recurrence, these molecular markers were analyzed in 400 formalin-fixed, paraffin-embedded PTC tissue specimens. The expression levels of miRNAs were measured using qRT-PCR. It was demonstrated that expression levels of all analyzed miRNAs are significantly higher in recurrent PTC than in non-recurrent PTC (*p* < 0.05). Moreover, higher expression levels of miR-146b, miR-222, miR-21, and miR-221 were associated with other clinicopathologic features of PTC, such as tumor size and lymph node metastases at initial surgery (*p* < 0.05). No significant differences in the frequency of BRAF^V600E^ mutation in recurrent PTC and non-recurrent PTC were determined. Our results suggest that miRNA expression profile differs in PTC that is prone to recurrence when compared to PTC that does not reoccur after the initial surgery while BRAF^V600E^ mutation frequency does not reflect the PTC recurrence status. However, the prognostic value of the analyzed miRNAs is rather limited in individual cases as the pattern of miRNA expression is highly overlapping between recurrent and non-recurrent PTC.

## 1. Introduction

Papillary thyroid carcinoma (PTC) is the most common type of thyroid cancer and it accounts for approximately 80% of thyroid cancer cases. For most PTC patients the prognosis is good, and the 5-year survival rate is about 90% [1]. Nevertheless, up to 10% of PTC patients after primary tumor removal and treatment suffer regional or distant metastatic recurrence [2]. Fine-needle aspiration biopsy (FNAB) is the most widely used method for presurgical diagnosis of PTC malignancy. However, this method has many limitations and is not very sensitive—for up to 20% FNAB diagnosis is unclear and surgery is needed to evaluate malignancy [3]. Therefore, the development of molecular markers of PTC recurrence such as miRNAs or BRAF^V600E^ mutation has the potential to improve the clinical management of patients by assisting in risk stratification.

MicroRNAs (miRNAs) are single-stranded, non-coding small RNAs that are usually 21–25 nucleotides in length. MiRNAs bind to the 3‘UTR region of the target mRNAs and suppress their translation by degradation or inhibition. In this way, these molecules regulate the expression of many oncogenes or tumor suppressor genes [4]. Dysregulation of miRNA expression is related to several cancer types, including PTC [5]. Previous studies have reported that some miRNAs (such as miR-21, miR-146b, miR-9, miR-221, miR-222, miR181b, miR-155, miR-220, and others) are dysregulated in PTC compared to healthy patients [6,7,8,9,10]. There were also several studies that analyzed the association between the miRNA expression and PTC recurrence, but the results were not consistent as they included only small groups of PTC patients [8,11,12,13]. Thus, it is still unclear whether specific miRNAs could be used as PTC recurrence biomarkers.

BRAF^V600E^ mutation is the transversion of thymine to adenine at nucleotide position 1799, therefore it leads to valine replacement by glutamic acid at codon 600. This mutation is the most common genetic lesion in PTC [14]. BRAF^V600E^ mutation is found in 45%–80% of PTC cases [15,16,17]. Some studies revealed that this mutation is associated with clinicopathologic parameters of poor outcome in PTC [18,19,20,21], however, others have not shown the association [22]. It is supposed that BRAF^V600E^ mutation might be related to miRNA expression in PTC but this association is still unclear. In previous studies, it was shown that the expression of some miRNAs is dysregulated in tissues with BRAF^V600E^ mutation [8,13,23] while others have not determined any alterations [13,24].

Despite low mortality rates, recurrence in PTC occurs in up to 10% of patients [2]. Currently, PTC recurrence risk stratification is accomplished by using clinicopathologic factors. Molecular markers, such as cyclin D1, p27, p21, osteopontin, and E-cadherin, have been studied, however, none of them have been approved as PTC recurrence markers [25,26]. Thus, personalized treatment of patients is hard to achieve. Identification of new specific biomarkers might improve diagnostics of PTC, recurrence risk stratification, and would help to optimize PTC treatment for individual patients. 

The aim of the current study was to identify specific miRNAs as biomarkers for predicting the recurrence of PTC. We determined the expression levels of five miRNAs (-146b, -222, -21, -221 and -181b) that were selected for this study based on previous reports [6,7,8,9,10] in a large number of paraffin-embedded PTC tissue samples. Additionally, we analyzed the association of miRNA expression levels with BRAF^V600E^ mutation and clinicopathologic characteristics of PTC. For the first time, the expression level of miR-181b in recurrent and non-recurrent PTC was analyzed.

## 2. Materials and Methods 

### 2.1. Human Tissue Samples

The formalin-fixed, paraffin-embedded (FFPE) PTC tissues (*n* = 400) were obtained from patients (aged from 18 to 83) who underwent total thyroidectomy at the Hospital of Lithuanian University of Health Sciences Kaunas Clinics between 2003 and 2017. After initial thyroid surgery all patients underwent postoperative radioactive iodine-131 (RAI) ablation and were treated with TSH-suppressive Levothyroxine doses. Patients were divided into two groups, depending on whether they had PTC recurrence after initial treatment or not. The non-recurrent group (NR-PTC, *n* = 313) comprised patients who had no signs of PTC recurrence after an initial treatment. They had undetectable levels of Tg and anti-Tg antibodies and a negative neck ultrasound after the initial surgery and RAI ablation at their latest follow-up. NR-PTC patients were selected randomly from the surgical database. Patients in the PTC recurrent group (Rc-PTC, *n* = 87) included those with a subsequent appearance of histologically confirmed PTC in the neck. The Rc-PTC group included only patients who had “true” PTC recurrence after the initial treatment. We defined disease as a “true” recurrence if a patient had local PTC recurrence at least 1 year after clinical and serological remission. Patients with persistent disease were not included in the study. All patients who had reoperative thyroid surgery due to recurrent PTC between 2003 and 2017 were included in the study.

The follow-up time for the patient was defined as the time period from the initial treatment to tumor recurrence or patient‘s death or, in the case of NR-PTC, to the most recent clinic visit. The follow-up time was censored in the disease-free survival analyses. 

The study was approved by the Kaunas Regional Committee of Biomedical Research (Lithuania, approval No. BE-2-44; 2015-12-23). Written informed consent was obtained from each participant of the study after full explanation of the purpose and nature of all procedures used. This study was conducted in accordance with the Declaration of Helsinki.

### 2.2. RNA and DNA Extraction

Genomic DNA and RNA were extracted from 5–10 mm^3^ sections of FFPE PTC tissues using the QIAamp DNA FFPE Tissue Kit (Qiagen, Hilden, Germany) and miRNeasy FFPE Kit (Qiagen, Hilden, Germany), respectively, according to the manufacturer’s protocols. The PTC tissue samples were macrodissected from areas that contained over >90% of malignant tissue. RNA and DNA concentration and quality were examined by NanoDrop 2000 Spectrophotometer (ThermoFisher Scientific, Waltham, MA USA).

### 2.3. BRAF^V600E^ Mutation Analysis

The sequence of BRAF gene encompassing codon 600 (fragment length 224 bp) was amplified by PCR. The composition of PCR mixture included Maxima Hot Start Master Mix (2×) (Thermo Fisher Scientific Baltics, Lithuania), forward (5′-TCA TAA TGC TTG CTC TGA TAG GA-3′) and reverse (5′-GGC CAA AAA TTT AAT CAG TGG A-3′) primers (10 μM), water and approximately 40 ng of purified genomic DNA. PCR reaction was run according to the following protocol: 95 °C for 5 min, 35 cycles: 95 °C 30 sec, 60 °C 1 min, 72 °C 1 min, and a final extension 72 °C 5 min. Amplification products were analyzed in ethidium bromide stained 2% agarose gel. Sequencing was performed by Applied Biosystems 3730xl DNA Analyzer (Baseclear, Leiden, The Netherlands).

### 2.4. qRT-PCR for miRNA Measurement

The expression levels of miRNAs were measured using quantitative reverse transcriptase-polymerase chain reaction (qRT-PCR) by TaqMan miRNA assays (Applied Biosystems, Foster City, Calif). Complementary DNA (cDNA) was synthesized from the purified total RNA using TaqMan miRNA Reverse Transcription Kit (Applied Biosystems, Foster City, Calif) following the manufacturer’s protocol. Real-time PCR was performed using TaqMan Universal PCR Master Mix (Applied Biosystems, Foster City, Calif) and Rotor-Gene 6000 PCR system (Corbett Research, Hilden, Germany). The fold expression changes were calculated by 2^-ΔΔCt^ method. Relative expression was calculated using 2^-ΔCt^ method where ΔCt is the difference in threshold cycles for target and reference genes. Firstly, purified RNA was reverse transcribed to cDNA using TaqMan miRNA primers according to the manufacturer’s instructions. As the starting material, 20 ng of tumor-derived RNA was used. Then the synthesized cDNA was amplified using TaqMan miRNA probes with the Rotor-Gene 6000 Real-time PCR System (Corbett Research, Germany). Three PCR reactions were run per sample according to the manufacturer’s instructions. We tested three endogenous controls for data normalization: RNU48, miRNA-16, and Let-7a. MiRNA Let-7a was chosen as an internal control for data normalization. As miRNA-181b expression level was very similar to miRNA Let-7a expression level and therefore had very small relative expression, in all presented figures miRNA-181b relative expression is plotted with a separate scale. Raw data of selected miRNA relative expression and clinical features is presented in Supplementary Table S1.

### 2.5. Statistical Analysis

Chi-square (χ2) test was used for analysis of data when variables were categorical. Continuous variables were tested for normality according to the Kolmogorov–Smirnov test. The results were presented as means ± standard deviation (SD) if the distributions were normal or median and range (min–max) if the distributions did not meet the criteria of normality.

Student’s *t*-tests (for normally distributed variables) or the Mann–Whitney tests (for non-normal distributions) were used to compare two groups of continuous variables. Multivariate binary logistic regression analysis was performed to estimate associations between different variables and PTC recurrence. 

Patients with miRNA expression below the median values were assigned as having low expression levels, and patients with miRNA above or equal to median assigned as having high expression levels. The association between miRNA expression levels, clinicopathological features and disease-free survival (DFS) were assessed by the Kaplan–Meier method. A log-rank test was used to estimate the statistical differences in Kaplan–Meier curves. Univariate Cox proportional hazard regression model was performed to evaluate significant clinopathological and molecular parameters for DFS. Multivariate Cox proportional hazard regression model with enter method was conducted to evaluate independent prognostic predictors for DFS.

Data were analyzed using STATISTICA 13.2, Microsoft Excel (2013), and IBM SPSS Statistics for Mac (V20.0). A *p* value less than 0.05 was considered as statistically significant.

## 3. Results

### 3.1. Characteristics of the Study Population

In total, 400 samples of FFPE PTC tissues were investigated in the study. Demographic and clinicopathological characteristics of the study population are presented in Table 1. The mean age of patients was 50 years and ranged from 18 to 83 years. More than three-quarters of patients were females, giving a ratio (male:female) of 1∶6.5. However, males made up a significantly higher percentage in the Rc-PTC group (23%) than in the NR-PTC group (10.6%), *p* = 0.003. NR-PTC consisted of 313 patients with a median age at initial surgery of 53 (18–83) years and Rc-PTC consisted of 87 patients with a median age of 47 (18–75) years (*p* < 0.001). Patients <45 years had higher frequency of PTC recurrence than patients ≥45 years, *p* = 0.001. Rc-PTC patients had more advanced tumor stage than NR-PTC patients. Moreover, 22.8% of the patients had regional lymph node metastases at the time of initial surgery with higher frequency in the Rc-PTC group than in the NR-PTC (49.4% vs. 15.4%, *p* < 0.001).

### 3.2. miRNA Expression Levels in PTC and Their Association With PTC Recurrence

FFPE PTC tissue samples collected for this study were stored for up to 15 years. The median age of FFPE PTC tissue samples were 9.92 (4.67–14.08) and 9.83 (5.5–14) years, respectively, for NR-PTC and Rc-PTC groups (*p* = 0.207). The yields of total RNA isolated from these samples varied from 0.36 to 13.65 μg with an average yield 2.75 μg per sample. The average A260/280 ratio for all samples was 1.7. Using the same input of RNA quantity, the Ct range for control miRNA let-7a varied from 16.8 to 23.9 cycles. 

**Figure 1:**
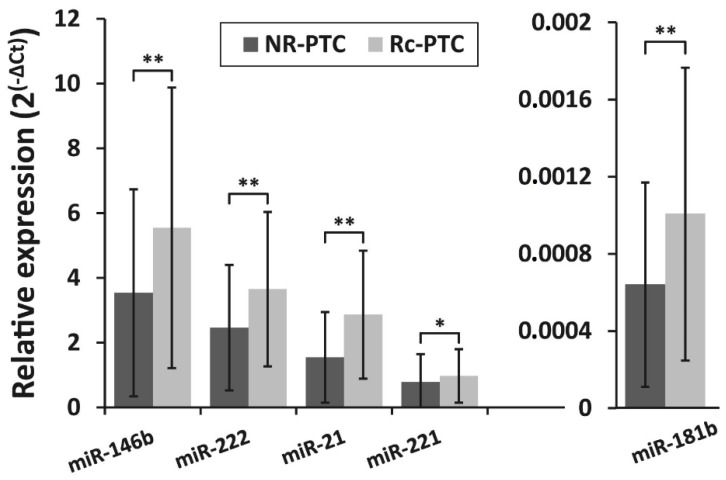
The relative expression levels of miR-146b, miR-222, miR-21, miR-221, and miR-181b in two patient groups—with recurrent papillary thyroid carcinoma (PTC) (Rc-PTC, *n* = 87) and without the recurrence of PTC (NR-PTC, *n* = 313). All data are presented as the mean ± SD. * *p* < 0.05, ** *p* < 0.01.

The expression levels of selected miRNAs (miR-146b, miR-222, miR-21, miR-221, miR-181b) were analyzed in Rc-PTC (*n* = 87) and NR-PTC (*n* = 313) groups. The expression levels of all miRNAs differed significantly between NR-PTC and Rc-PTC groups (*p* ˂ 0.05). MiR-146b, miR-222, miR-21, miR-221, and miR-181b were overexpressed 1.6-fold (*p* ˂ 0.01), 1.5-fold (*p* ˂ 0.01), 1.9-fold (*p* ˂ 0.01), 1.2-fold (*p* = 0.01), and 1.6-fold (*p* ˂ 0.01), respectively, in Rc-PTC compared to NR-PTC. Relative expression levels of all five miRNAs in NR-PTC and Rc-PTC groups are shown in Figure 1. As shown in this graph, there are substantial overlaps between miRNA profiles in NR-PTC and Rc-PTC groups. The overlap of miRNA expression profiles in Rc-PTC and NR-PTC groups can also be seen in Supplementary Figure S1. Although the samples with higher miRNA expression are more frequent in the Rc-PTC group than in the NR-PTC group this difference is too small to identify patients with high risk of PTC recurrence. ROC curve analysis also showed that all analyzed miRNAs are relatively weak biomarkers to stratify patients between Rc-PTC and NR-PTC groups (AUCs values for all miRNAs is less than 0.8) (Supplementary Figure S2).

### 3.3. Detection of BRAF^V600E^ Mutation in PTC Recurrence and Non-Recurrence Groups and Its Association With miRNA Expression

In total, 205 PTC samples were analyzed for BRAF^V600E^ mutation by PCR and sequencing. The frequency of BRAF^V600E^ mutation was analyzed in two patient groups: NR-PTC (*n* = 135) and Rc-PTC (*n* = 70). BRAF^V600E^ mutation was detected in 62% of all PTC samples. Comparing NR-PTC and Rc-PTC groups, the frequency of BRAF^V600E^ mutation was not significantly different (*n* = 83 (61.5%) and *n* = 44 (62.9%), *p* = 0.847, respectively).

**Figure 2:**
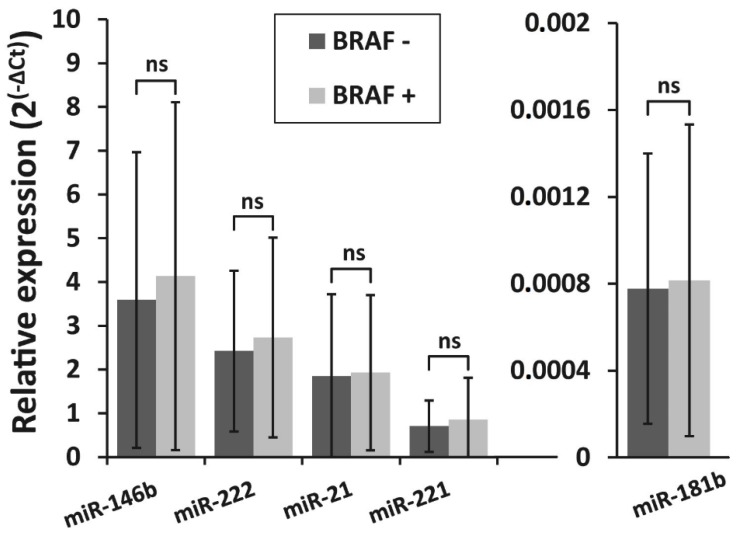
Relative expression levels of miR-146b, miR-222, miR-21, miR-221, and miR-181b in two PTC groups—BRAF^V600E^-positive (BRAF +, *n* = 127) and BRAF^V600E^ -negative (BRAF −, *n* = 78). All data are presented as the mean ± SD. ns—not significant.

The correlation between BRAF^V600E^ mutation and the expression levels of all five selected miRNAs was investigated in PTC samples. All samples were divided into two groups: BRAF^V600E^-positive (BRAF +; *n* = 127) and BRAF^V600E^-negative (BRAF −; *n* = 78). The expression levels of all miRNAs did not differ significantly between BRAF^V600E^-positive and BRAF^V600E^-negative PTC groups (*p* > 0.05) (Figure 2).

### 3.4. Association of miRNAs Expression and BRAF^V600E^ Mutation with Clinicopathologic Features of PTC

**Figure 3:**
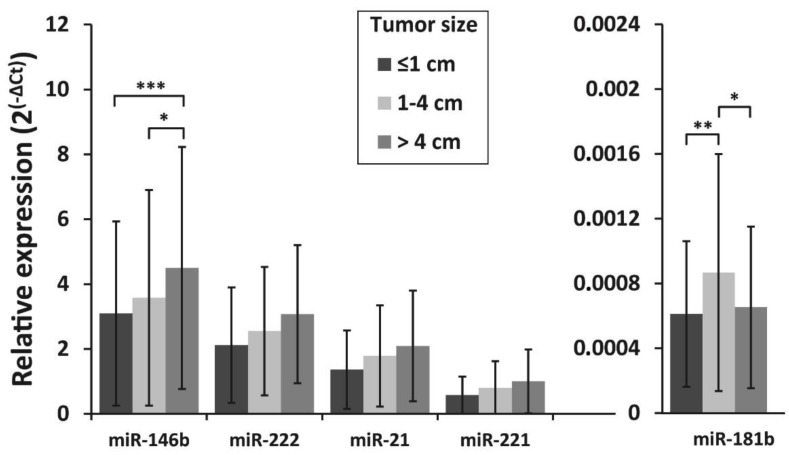
Relative expression levels of miR-146b, miR-222, miR-21, miR-221, and miR-181b in differently sized PTC tumors. All data are presented as the mean ± SD. * *p* < 0.05, ** *p* < 0.01, and *** *p* < 0.001.

The association of miRNA expression with tumor size, lymph node metastasis status at initial surgery, and patient’s age was also investigated. To evaluate the association between tumor size and miRNA expression levels, all samples were divided into three groups according to the primary tumor size: tumors 1 cm or less in size (microcarcinomas) (*n* = 109), tumors >1–≤4 cm (*n* = 109) and tumors >4 cm (*n* =182). MiR-146b expression levels were similar between patients with PTC microcarcinoma and those who had tumors >1–≤4 cm (*p* = 0.405), however, a significantly higher expression of miR-146b was found in tumors >4cm than in tumors >1–≤4 cm or tumors ≤1cm (*p* = 0.012 and *p* < 0.001, respectively). For miR-181b, higher expression levels were found in tumors >1–≤4 cm than in smaller or larger tumors (*p* = 0.005 and *p* = 0.014, respectively) (Figure 3).

**Figure 4:**
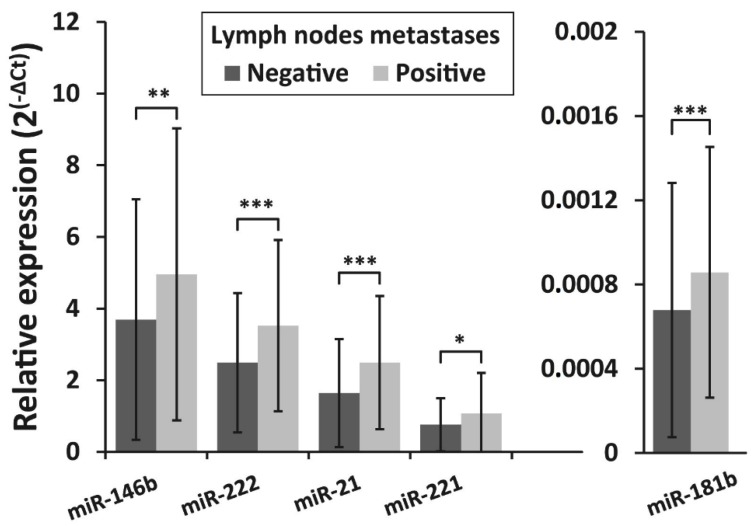
Relative expression levels of miR-146b, miR-222, miR-21, miR-221, and miR-181b in PTC in patients with lymph node metastases at initial surgery and patients without metastases. All data are presented as the mean ± SD. * *p* < 0.05, ** *p* < 0.01, and *** *p* < 0.001.

The expression levels of all investigated microRNAs were found to be associated with lymph node metastasis status at initial surgery. Expression levels of miR -146b, miR -222, miR -21, miR-221, and miR-181b were significantly higher in the lymph node metastases-positive group (*n* = 91) compared to the lymph node metastases-negative group (*n* = 309) (*p* = 0.004, *p* <0.001, *p* <0.001, *p* = 0.012, and *p* < 0.001, respectively) (Figure 4).

**Figure 5:**
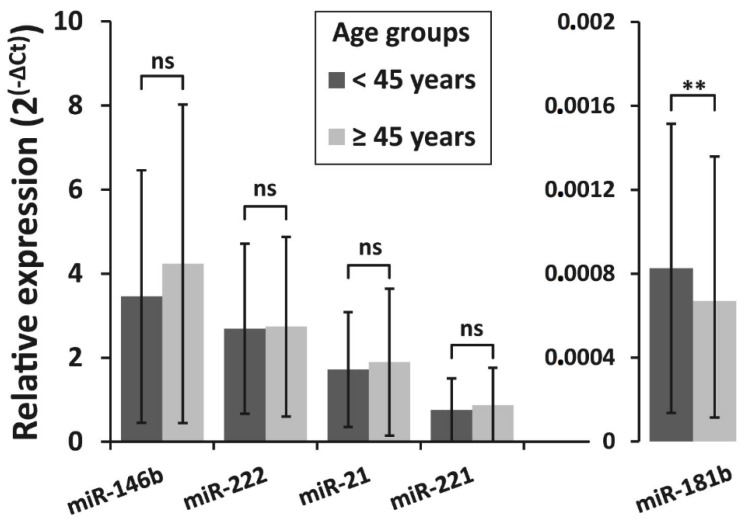
Relative expression of miR-146b, miR-222, miR-21, miR-221, and miR-181b in different age groups of PTC patients. All data are presented as the mean ± SD. ** *p* < 0.01. ns—not significant.

Furthermore, patients were divided into two age groups: younger than 45 years at PTC diagnosis (*n* = 132) and 45 years and older (*n* = 268). Younger patients had similar expression levels of miR-146b, miR-222, miR-21, and miR-221 as compared to older patients (*p* = 0.11, *p* = 0.944, *p* = 0.485, *p* = 0.314, respectively). Only miR-181b levels were associated with patient’s age at PTC diagnosis. Higher expression of miR-181b was found in patients younger than 45 years compared to older patients (*p* = 0.007) (Figure 5).

**Figure 6:**
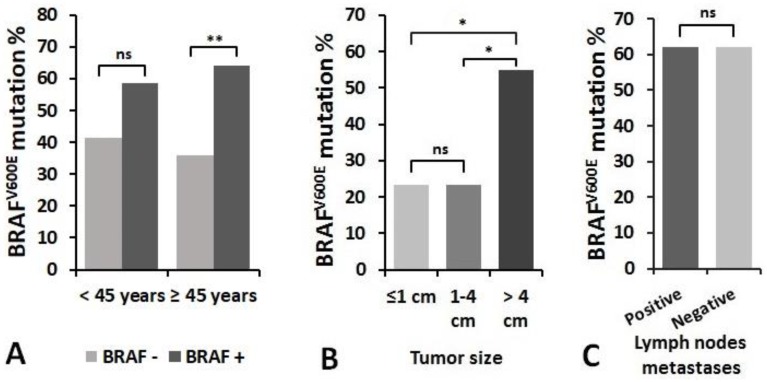
The relationship between the frequency of BRAF^V600E^ mutation and clinicopathological features of PTC: (**A**) patient’s age at diagnosis, (**B**) tumor size, and (**C**) the presence of regional lymph node metastases at initial surgery. * *p* < 0.05, ** *p* < 0.01. ns—not significant.

**Figure 7:**
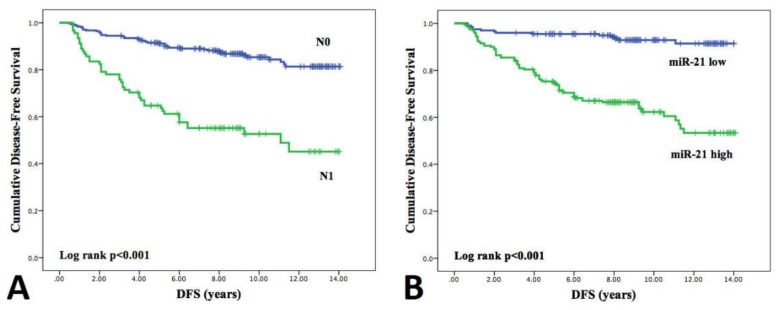
Kaplan–Meier curves estimating disease-free survival according to (**A**) the presence of lymph node metastasis and (**B**) high/low expression levels of miR-21. DFS curves were compared using the Log rank test.

The association of BRAF^V600E^ mutation with patient’s age at diagnosis of PTC, primary tumor size and lymph node metastasis status were investigated (Figure 7). Patients of 45 years and older had the same frequency of BRAF^V600E^ mutation as younger patients, whereas the significant difference was found in analysis limited within age groups. In 45 years and older patients group, a higher proportion of patients had BRAF^V600E^ mutation (64.2% vs. 35.8%, *p* =0.002), whereas in the younger patients group the BRAF^V600E^ positivity group did not significantly differ (58.5% vs. 41.5%, *p* = 0.122) (Figure 6A).

When assessing the relationship between BRAF^V600E^ mutation and tumor size, samples were divided into three groups: tumor size ≤1 cm (*n* = 51), tumor size >1–≤4 cm (*n* = 56), and tumors >4 cm in size (*n* = 98). We found that BRAF^V600E^ mutation occurred more frequently in a group of patients with primary tumor >4 cm in size compared to smaller tumors (*p* < 0.05) (Figure 6B). There was no significant relationship between BRAF^V600E^ positivity and the presence of regional lymph nodes metastases at initial surgery. BRAF^V600E^ mutation was detected in 62.0% of samples in the metastasis group (*n* = 31) and in 61.9% of samples in the group without metastases (*n* = 96) (*p* = 0.993) (Figure 6C).

The multivariate logistic regression analysis was performed to select the independent prognostic parameters for PTC recurrence. The analysis showed that positive lymph nodes and miR-21 were independently associated with elevated odds of PTC recurrence, OR 3.66 (95% CI 1.59–8.45) and OR 1.50 (95% CI 1.12–2.00), respectively (Table 2).

### 3.5. Influence of Clinicopathological Features and miRNA Expression on Disease-Free Survival (DFS)

The median follow-up time in NR-PTC group was 9 (3.08–14.08) years and median time to relapse in Rc-PTC group was 3.25 (1.17–11.5) years. To evaluate DFS association with miRNA expression levels patients were divided into high and low miRNAs expression groups. Univariate Cox regression hazards model analysis was performed that included clinicopathological features, BRAF^V600E^ mutation status, and the expression levels (high/low) of each investigated miRNA (Table 3). Analysis revealed that females and patients with microcarcinomas have significantly lower hazard of recurrence, while younger patients, presence of metastatic lymph nodes, and high expression levels of all selected miRNAs were associated with increased hazard of shorter DFS. Variables that were chosen for inclusion in the multivariate analysis were those with significant univariate associations with DFS in this study, as well as those (i.e., BRAF^V600E^) found in other studies to be significant predictors for DFS. After multivariate Cox proportional regression hazard model analysis, only metastatic lymph nodes and high miR-21 expression level emerged as independent prognostic factors associated with shorter DFS (HR 1.94 (95% CI 1.12–3.36) and HR 5.84 (95% CI 2.79–12.28), respectively).

Kaplan–Meier plots for independent prognostic factors are reported in Figure 7 and for the remaining factors are presented in Supplementary Figure S3. The Log-rank test demonstrated significant differences in survival curves both for lymph node metastases (*p* < 0.001) and miRNA-21 expression (*p* < 0.001). Five-year disease-free survival was 92.1% in patients with negative lymph nodes and 65.7% in patients with metastatic lymph nodes, and 95.3% in patients with low miR-21 expression levels and 75.4% in patients with high miR-21 expression. 

## 4. Discussion

In this study, we investigated five selected miRNAs (-146b, -222, -21, -221, and -181b) as biomarkers for predicting PTC recurrence and analyzed the associations of miRNAs expression with BRAF^V600E^ mutation and clinicopathologic characteristics of PTC. Five miRNAs (-146b, -222, -21, -221, and -181b) were selected for this study based on previous reports [6,7,8,9,10]. Our investigation is the largest study as yet. We investigated 400 PTC samples, meanwhile, other studies involved fewer than 221 samples [8,11,12,24,28,29,30]. Most miRNAs are known to be very stable in tissue specimens making them a suitable marker to be analyzed in archived FFPE samples [31]. However, not much is known about the stability of widely used non-miRNA endogenous controls. It is likely that they are more prone to degradation because of their length and different functions in the cell. In the current study, we have tested three previously published endogenous controls for data normalization: RNU48 [8,10,11,24,28], miRNA-16 [2], and Let-7a [9,32]. When comparing the assay performance with these different controls, we have noticed that the commonly used endogenous control RNU48 degrades significantly faster in archived FFPE samples than any of our analyzed target miRNAs (our unpublished data). In contrast, Let-7a had similar stability as the target miRNAs. Therefore, we chose to use Let-7a as an endogenous control which was also used in several similar studies [9,32]. 

In our study, we first compared the expression profile of selected miRNA in PTC in general with miRNA expression in healthy thyroid tissue and demonstrated that four of five analyzed miRNAs (miR-146b, miR-222, miR-21, and miR-221) were significantly overexpressed in PTC. These results were in agreement with other reports [7,9,10,33,34]. Next, we divided PTC samples according to the PTC recurrence status and determined that expression levels of miR-146b, miR-222, miR-21, miR-221, and miR-181b were significantly higher in PTC recurrence group as compared to the non-recurrence group (*p* < 0.05). In line with these data, previous studies in smaller-sized groups of PTC patients have revealed enhanced expression of certain miRNAs in recurrent PTC [8,11,12,28,29,30]. In previous studies, the difference of miR-181b expression levels between Rc-PTC and NR-PTC groups has not been investigated. For the first time, we analyzed the association of miR-181b expression with PTC recurrence status and demonstrated different expression levels of miR-181b in NR-PTC and Rc-PTC groups (*p* < 0.05). In the present study, we found that metastatic lymph nodes at the time of diagnosis and expression of miR-21 are associated with PTC recurrence and were independent prognostic factors of DFS.

Although the levels of all studied miRNAs were significantly higher in the Rc-PTC group, an overlap of miRNA expression profiles in NR-PTC and Rc-PTC groups with a small difference between the peaks was observed. This indicates that the analyzed miRNA does not represent reliable prognostic markers that would allow identifying patients with a high risk of PTC recurrence. This observation was also confirmed by the ROC curve analysis. The AUCs for miR-146b, miR-222, miR-21, miR-221, and miR-181b were 0.685, 0.676, 0.762, 0.598, and 0.685, respectively.

BRAF^V600E^ mutation is the most common genetic lesion in PTC [14]. In this study, the frequency of BRAF^V600E^ mutation was investigated in 221 PTC samples. BRAF^V600E^ mutation was detected in 61.5% of all PTC samples. In other studies, BRAF^V600E^ mutation was identified in 45–80% of PTC cases [13,15,16,17,24]. Comparing NR-PTC and Rc-PTC groups, the frequency of BRAF^V600E^ mutation was not significantly different (*p* = 0.641). There are limited data on the association of BRAF^V600E^ mutation and PTC recurrence. We found only one study where the frequency of BRAF^V600E^ mutation was analyzed in NR-PTC and Rc-PTC groups. In line with our study, it was reported that the frequency of BRAF^V600E^ mutation was not different between NR-PTC and Rc-PTC groups (*p* > 0.05) [8].

The associations of BRAF^V600E^ mutation with clinicopathological features were also investigated. Study results revealed that tumor size >4 cm significantly associated with BRAF^V600E^ mutation (*p* < 0.05). However, lymph node metastasis was not significantly different between patients with or without BRAF^V600E^ mutation (*p* = 0.993). In previous reports, there are limited and controversial data on the association of BRAF^V600E^ mutation with patient age, tumor size, and metastasis status. In line with our data, some studies demonstrated statistically significant correlations between BRAF^V600E^ mutation and patient age [19,35,36,37,38,39], between BRAF^V600E^ mutation and tumor size [24,35,39,40,41,42], and between BRAF^V600E^ mutation and regional lymph nodes metastases [35,39,40,41,42]. However, other studies did not find a statistically significant correlation between BRAF^V600E^ mutation and patient age [13,43,44,45,46], tumor size [13,22,37,47], and metastasis status [13,36,43]. We also did not find any significant association between BRAF^V600E^ mutation and miRNA expression, which is in line with previous reports [24]. BRAF^V600E^ usefulness as a prognostic marker in PTC remains controversial. Many previous studies reported a higher risk of PTC recurrence, metastases, and poor disease outcome in patients with BRAF^V600E^ mutation, although there are plenty of studies that could not confirm any mutation-related associations [48,49,50]. These authors found that other factors, such as the presence of lymph node metastases, extrathyroidal invasion, or histologic type of PTC, provide higher accuracy in predicting disease outcome than BRAF mutation status alone [49,50,51]. 

Although miRNA expression profiles in PTC have been analyzed for more than a decade, little is known regarding the genes and pathways downstream that are regulated by miR-146b, miR-222, miR-221, miR-21, and miR-181b as only a few computationally predicted targets are validated in laboratory experiments. MiR-21 is one of the most extensively studied miRNAs in cancer and it was one of the first miRNAs described as a potential tumor biomarker. It is highly conserved across many species and its overexpression has been detected in many cancer types suggesting miR-21 role in gene regulation which disruption may contribute to cancer development. Studies with various cancer cell lines also showed that eliminating miR-21 expression inhibits the development of cancer-associated phenotypes. Moreover, IL-6, STAT3, AP-1, and TGFβ1 were described as molecules involved in the upregulation of miR-21 expression [52]. In the present study, we found that high levels miR-21 both with metastatic lymph nodes at the time of diagnosis are associated with PTC recurrence and were independent prognostic factors of DFS.

Another well-studied miRNA is miR-146b. Some of the detected downstream targets of miR-146b are SMAD4 and IRAK1 whose downregulation leads to an increased proliferation and migration activity and inhibits cell cycle arrest in PTC cell line [53,54]. MiR-221 and miR-222 are two highly homologous miRNAs that are also overexpressed in many types of cancer including PTC. The determined targets of these miRNAs are p27, p57, and PTEN which all have important roles in cell cycle regulation [55]. Among all dysregulated miRNAs in cancer, miR-181b has been found to be a critical regulatory miRNA linking inflammation and cancer by suppressing cylindromatosis expression. The expression of miR-181b is regulated by STAT3 and HMGA1 [56]. The functional significance of miR-146b, miR-222, miR-221, miR-21, and miR-181b in various tumors suggests that they exhibit great potential as predictive and prognostic biomarkers. Extensive efforts are underway to identify mRNA targets and the affected regulatory networks, which may be the key to providing a better understanding of PTC development and miRNA role in carcinogenesis in general.

## 5. Conclusions

Summarizing, the analysis of five miRNA (-146b, -222, -221, -21, and -181b) expression levels in a large number of well-characterized PTC samples (*n* = 400) revealed that these miRNAs are not only overexpressed in PTC, but they are also overexpressed in PTC with more aggressive features such as recurrence, bigger tumor size, lymph node metastasis, and shorter disease-free survival. However, the prognostic value of these miRNAs is rather limited in individual cases as the distribution of miRNA expression overlaps between patients with high and low risk of PTC recurrence. Additionally, more standardization is needed on tissues and different endogenous controls used in miRNA research as these factors have a significant impact on the final results. Different approaches such as analysis of circulating miRNAs, search for another more reliable molecular markers, or single-cell analysis might represent the future trends for improving the recognition of patients with recurrent PTC.

## Figures and Tables

**Table 1:** Demographic and clinicopathological characteristics of the study population.

Characteristic	All Patients (*n* = 400)	Non-Recurrence Group (*n* = 313)	Recurrence Group (*n* = 87)	*p*
Sex, *n* (%) Male Female	53 (13.3) 347 (86.7)	33 (10.6) 280 (89.4)	20 (23.0) 67 (77.0)	**0.003**
Age at initial surgery (years) Median (min-max) <45 years, *n* (%) ≥45 years, *n* (%)	50 (18-83) 131 (32.8) 269 (67.2)	53 (18-83) 91 (28.8) 222 (71.2)	47 (18-75) 41 (47.1) 46 (52.9)	**<0.001** **0.001**
Tumor size *, *n* (%) pT1a pT1b pT2 pT3 pT4	109 (27.3) 81 (20.3) 23 (5.8) 172 (43.0) 15 (3.8)	97 (30.8) 64 (20.5) 18 (5.8) 128 (41.0) 6 (1.9)	12 (13.8) 17 (19.5) 5 (5.7) 44 (50.6) 9 (10.3)	**<0.001** **<0.001** **0.007** **<0.001** 0.439
Lymph node metastases at initial surgery, *n* (%)	91 (22.8)	48 (15.4)	43 (49.4)	**<0.001**

* Classification according to World Health Organization Classification of Tumors Pathology and Genetics of Tumors of Endocrine Organs [27].

**Table 2:** Multivariate analysis of associations of PTC recurrence with prognostic factors. Binary logistic regression model was used. Odds ratios adjusted by gender and age categories (<45 years vs. ≥45 years).

Variable	OR (95% CI)	*p*-Value
Tumor size (T1 vs. T2–4)	1.17 (0.56–2.44)	0.680
Lymph nodes (pos vs. neg)	3.66 (1.59–8.45)	0.002
BRAF^V600E^ (pos vs. neg)	1.01 (0.45–2.11)	0.994
miR- 146b	1.09 (0.93–1.27)	0.279
miR-222	1.21 (0.87–1.68)	0.248
miR-21	1.50 (1.12–2.00)	0.006
miR-221	0.86 (0.48–1.54)	0.862
miR-181b	1.08 (0.81–1.43)	0.618

**Table 3:** Univariate and multivariate analysis of disease-free survival (DFS) in patients with PTC. Cox proportional regression hazard model was used.

**Univariate Analysis** **DFS**		
	**HR (95% CI)**	*p*-Value
Gender (female vs. male)	0.47 (0.29-0.78)	0.003
Age (<45 years vs. ≥45 years)	1.77 (1.16-2.7)	0.008
Tumor size (T1 vs. T2-4)	0.52 (0.33-0.83)	0.004
Lymph nodes (pos vs. neg)	4.24 (2.78-6.47)	<0.001
BRAF^V600E^ (pos vs. neg)	1.01 (0.62-1.64)	0.964
miR-146b (high vs. low)	2.62 (1.65-4.15)	<0.001
miR-222 (high vs. low)	2.39 (1.52-3.77)	<0.001
miR-21 (high vs. low)	6.45 (3.63-11.44)	<0.001
miR-221 (high vs. low)	1.82 (1.76-2.79)	0.007
miR-181b (high vs. low)	3.15 (1.93-5.15)	<0.001
**Multivariate Analysis** **DFS**		
	HR (95% CI)	*p*-Value
Gender (female vs. male)	0.85 (0.46-1.56)	0.603
Age (<45 years vs. ≥45 years)	1.37 (0.83-2.26)	0.214
Tumor size (T1 vs. T2-4)	1.19 (0.7-2.04)	0.523
Lymph nodes (pos vs. neg)	1.94 (1.12-3.36)	0.017
BRAF^V600E^ (pos vs. neg)	0.8 (0.47-1.36)	0.410
miR-146b (high vs. low)	1.49 (0.77-2.89)	0.233
miR-222 (high vs. low)	1.03 (0.51-2.08)	0.94
miR-21 (high vs. low)	5.84 (2.79-12.28)	<0.001
miR-221 (high vs. low)	1.51 (0.89-2.57)	0.131
miR-181b (high vs. low)	1.32 (0.75-2.33)	0.340

## Supplementary Materials

The following are available online at https://www.mdpi.com/2218-273X/10/4/625/s1, Figure S1: Frequency distribution of (A) miR-146b, (B) miR-222, (C) miR-21, (D) miR-221, (E) miR-181b expression in NR-PTC and Rc-PTC groups, Figure S2: ROC curve analysis of miR-146b, miR-222, miR-21, miR-221, miR-181b for the stratification of patients who experienced PTC recurrence (Rc-PTC group) from patients who did not (NR-PTC group). The AUCs for miR-146b, miR-222, miR-21, miR-221 and miR-181b were 0.685, 0.676, 0.762, 0.598 and 0.685, respectively, Figure S3: Kaplan-Meier analysis of disease-free survival (DFS) according to the clinicopathological features, BRAF mutation, high/low expression levels of miRNA’s in patient’s cancer tissue: (A) Gender, (B) Age, (C) Tumor size, (D) BRAF mutation, (E) miR-146b, (F) miR-222, (G) miR-221, (H) miR-181b. DFS curves were compared using Log rank test, Table S1: Raw data of selected miRNA relative expression and clinical features.
