# Supplementary material for: Association of microRNA Expression and BRAFV600E Mutation with Recurrence of Thyroid Cancer
Source: Biomolecules. 2020 Apr 17;10(4):625. doi: 10.3390/biom10040625 (PMC7226510; doi:10.3390/biom10040625)
Supplement: Supplementary file 1 [file biomolecules-10-00625-s001.zip › Supplementary Figures.docx]

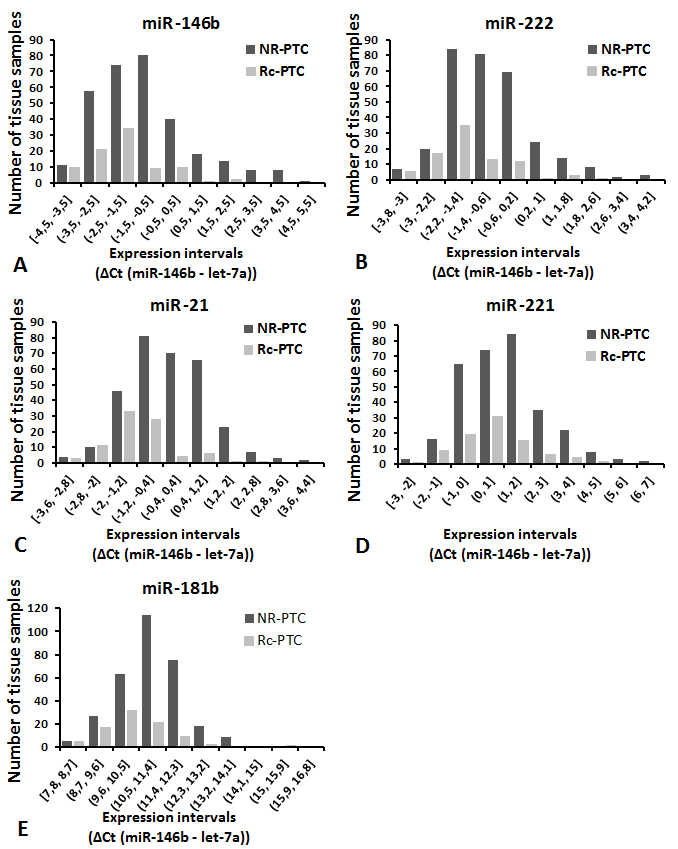


**Supplementary Figure 1.** Frequency distribution of (A) miR-146b, (B) miR-222, (C) miR-21, (D) miR-221, (E) miR-181b expression in NR-PTC and Rc-PTC groups.


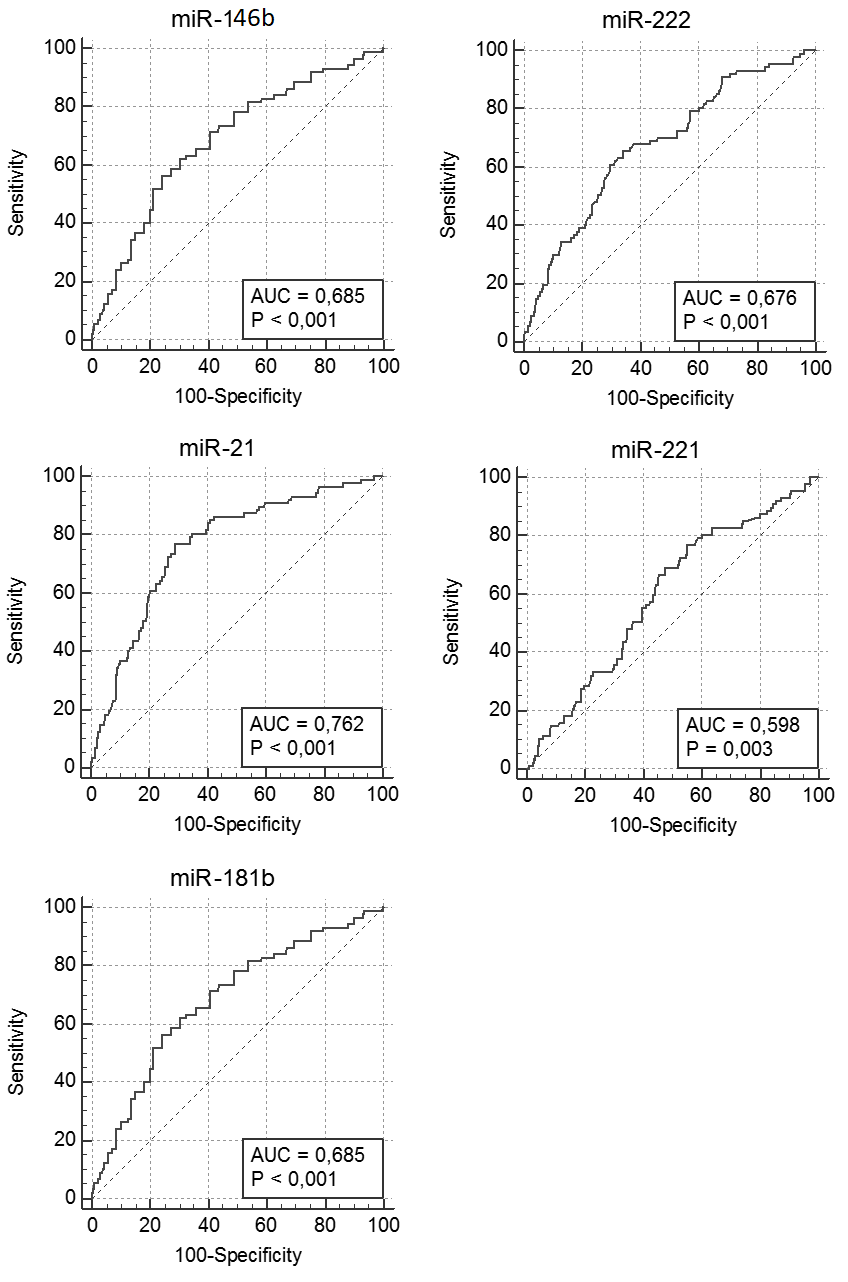


**Supplementary Figure 2.** ROC curve analysis of miR-146b, miR-222, miR-21, miR-221, miR-181b for the stratification of patients who experienced PTC recurrence (Rc-PTC group) from patients who did not (NR-PTC group). The AUCs for miR-146b, miR-222, miR-21, miR-221 and miR-181b were 0.685, 0.676, 0.762, 0.598 and 0.685, respectively.


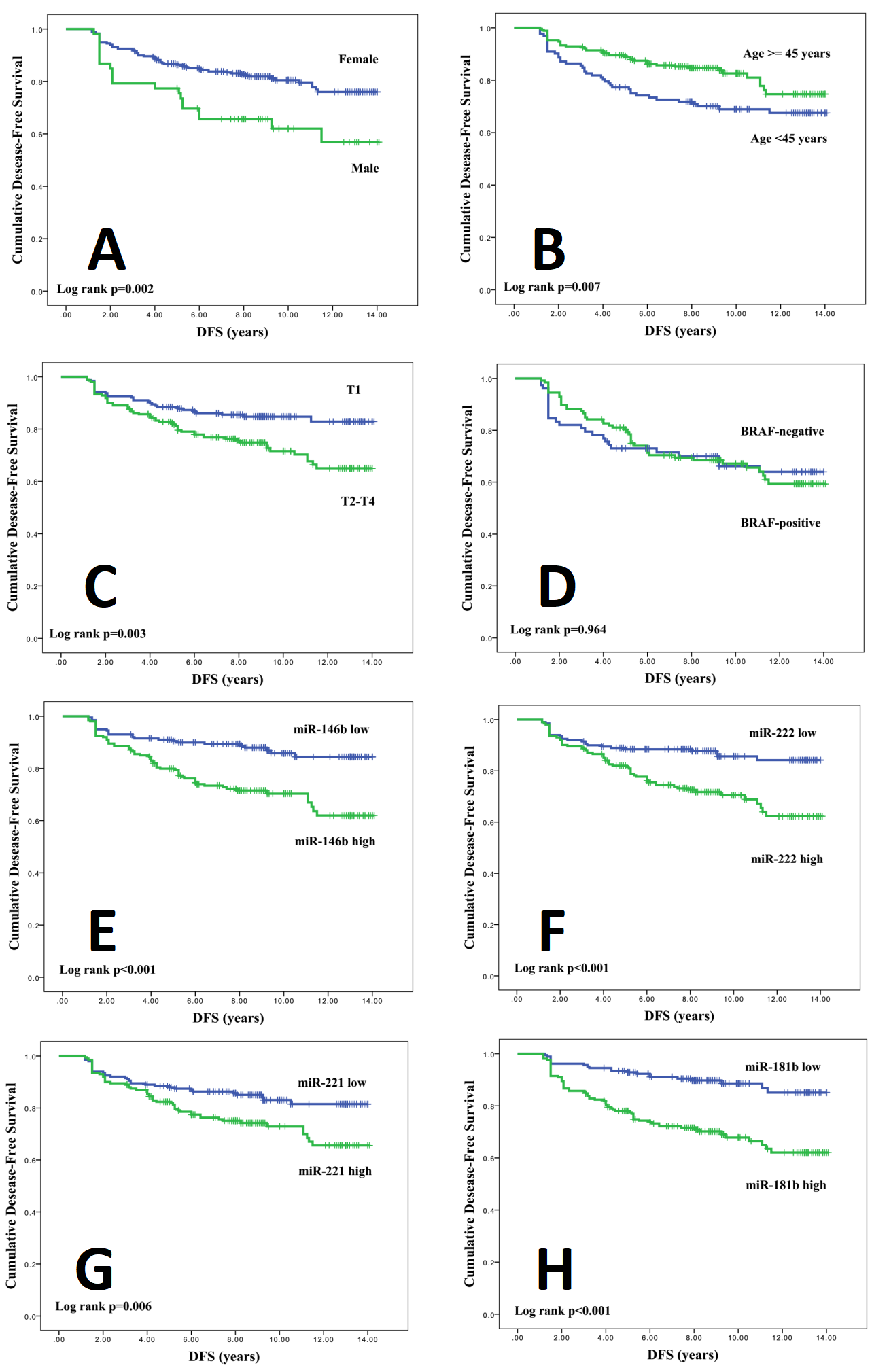


**Supplementary Figure 3.** Kaplan-Meier analysis of disease-free survival (DFS) according to the clinicopathological features, BRAF mutation, high/low expression levels of miRNA's in patients cancer tissue : (A) Gender, (B) Age, (C) Tumor size, (D) BRAF mutation, (E) miR-146b, (F) miR-222, (G) miR-221, (H) miR-181b. DFS curves were compared using Log rank test.
